# Exact and Effective Pair-Wise Potential for Protein-Ligand Interactions Obtained from a Semiempirical Energy Partition

**DOI:** 10.3390/ijms9091652

**Published:** 2008-09-02

**Authors:** Alexandre R. F. Carvalho, André T. Puga, André Melo

**Affiliations:** 1 REQUIMTE/Department of Chemistry, Faculty of Science, University of Porto, Rua do Campo Alegre, 687, 4169-007 Porto, Portugal. E-Mail: afcarval@fc.up.pt (A. C.); 2 DEMEGI, Faculty of Engineering, University of Porto, Rua Dr Roberto Frias, 4200-465 Porto, Portugal. E-Mail: apuga@fe.up.pt (A. P.)

**Keywords:** Protein-ligand interactions, energy partition scheme, association processes, stabilization energy, quantum partition of molecules, semiempirical methods

## Abstract

In this work, the partition method introduced by Carvalho and Melo was used to study the complex between *Cucurbita maxima* trypsin inhibitor (CMTI-I) and glycerol at the AM1 level. An effective potential, combining non-bonding and polarization plus charge transfer (PLCT) terms, was introduced to evaluate the magnitude of the interaction between each amino acid and the ligand. In this case study, the nonbonding–PLCT non-compensation characterizes the stabilization energy of the association process in study. The main residues (Gly29, Cys3 and Arg5) with net attractive effects and Arg1 (with a net repulsive effect), responsible by the stability of protein-ligand complex, are associated with large nonbonding energies non-compensated by PLCT effects. The results obtained enable us to conclude that the present decomposition scheme can be used for understanding the cohesive phenomena in proteins.

## 1. Introduction

Computational methods are of great interest to evaluate binding affinities between proteins and ligands, with many applications in structure-based drug design (SBDD) [1]. A complete description of the correspondent molecular interactions, including the short-range polarization plus charge transfer (PLCT) effects, can only be carried out at a quantum mechanics (QM) level. However, the more accurate QM methods require very large computational resources and this has limited high-level theoretical studies in this field [2]. Current methods using classical potentials can only represent a good approximation for evaluating the nonbonding protein-ligand interactions, because they are usually designed to treat the QM effects in an average manner. For this purpose, QM methods have been currently used to parameterize force fields [3–7] and scoring functions [8]. This has enabled computationally intensive SBDD studies at lower theoretical levels. The hybrid quantum mechanics/molecular mechanics (QM/MM) methods are an interesting alternative approach for this type of problems, because they conjugate an appropriate description of the molecular system with moderate computational resources [9, 10]. Good QM/MM methods require a rigorous partition of the molecular systems into two regions: a strongly perturbed region that should be described at a quantum level and a bulk region whose interactions can be reproduced by classical potentials. In this context, the energy partition schemes [11–13] can provide rational criterions to define these regions. *In vitro* methods and mathematical models for enzymatic reactions [14–16] are the prototype for evaluate kinetics and binding affinities between proteins and ligands.

In this work, the application of the SemiEmpirical Energy Based (SEEB) partition method introduced by Carvalho and Melo [17] to study protein-ligand association processes was analyzed. This method enables the stabilization energy decomposition both into physically meaningful and spatial components. As this formalism was developed at a semiempirical quantum level, it enables also the complete separability of these components. Here, the SEEB formalism was extended to describe protein-ligand interactions using a pair-wise potential. The SEEB method was then used to study the association between the Curcubita maxima trypsin inhibitor (CMTI-I) and glycerol. CMTI-I is well known by its biological importance [18, 19]. Glycerol is a cryoprotectant [20], which should be washed away with solvent in the crystallization process. However, it forms a stable complex with CMTI-I that is detected in the crystallized structure. In this context, glycerol should be considered to have a large affinity to CMTI-I and this study can provide a further insight for a rational modeling of high-specific ligands for this protein.

## 2. Methods

A non-covalent (no) association between a protein (P) with n amino acid residues and a ligand (L) can be represented by equation (1):

(1) P+L⇌P:L

**Figure 1.**
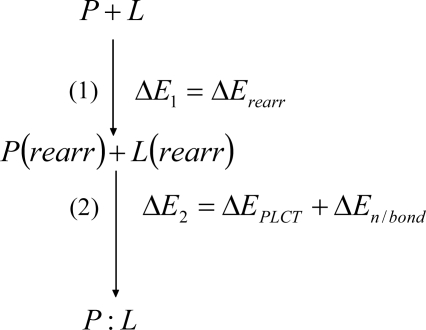
Hypothetical mechanism for a non-covalent association between a protein (P) and a ligand (L), which enables the application of SEEB formalism to partition the associated stabilization energy into physically meaningful components.

A general association process can be described by a hypothetical mechanism involving two steps (see Figure 1). In the first step, the molecular monomers (P and L) are rearranged assuming the geometries adopted in the dimer.

In the second step, the rearranged species (P(rearr) and L(rearr)) associate each other preserving their geometries and originating the dimer (P:L). According to the SEEB formalism, the stabilization energy can be partitioned as:

(2) $$\Delta E^{no}\;=\;\Delta E_{1}^{no}\;+\;\Delta E_{2}^{no}$$

In equation (2), the conformational rearrangement component ( $\Delta E_{rearr}^{no}$) is associated with step 1, while the polarization plus charge transfer ( $\Delta E_{PLCT}^{no}$) and nonbonding ( $\Delta E_{n/bond}^{no}$) components are associated with step 2:

(3) $$\Delta E_{1}^{no}\;=\;\Delta E_{rearr}^{no}\;=\;\Delta E_{rearr}^{no}(P)\;+\;\Delta E_{rearr}^{no}(L)$$

(4) $$\Delta E_{2}^{no}\;=\;\Delta E_{PLCT}^{no}\;+\;\Delta E_{n\;/\;bond}^{no}$$

In equations (2) to (4), the superscript (no) indicates that the P:L association is of non covalent nature. The nomenclature used for binding states is presented elsewhere [17]. The exact definition of the terms occurring in equation (3) and (4) is presented in appendix A.

An effective pair-wise potential, combining non-bonding and PLCT contributions, is proposed in this work:

(5) $$\Delta E_{eff}^{no}\;=\;\sum_{A=1}^{n}\Delta E_{eff}^{no}(AL)$$

In this context, the equation (4) can be rewritten as:

(6) $$\Delta E_{2}^{no}=\Delta E_{eff}^{no}$$

The transformation of the exact interaction energy decomposition (equations (2) to (3)) into the effective pair-additive expression (5) is developed and justified in Appendix B.

In consistency with the SEEB formalism, the stabilization energy associated with step 2 can be also partitioned into strongly perturbed ( $\Delta E_{pert}^{no}$) and bulk ( $\Delta E_{bulk}^{no}$) components:

(7) $$\Delta E_{2}^{no}=\Delta E_{pert}^{no}+\Delta E_{bulk}^{no}$$

Both components can then be partitioned into long-range nonbonding and short-range PLCT terms:

(8) $$\Delta E_{pert}^{no}=\Delta E_{pert,n/bond}^{no}+\Delta E_{pert,PLCT}^{no}$$

(9) $$\Delta E_{bulk}^{no}=\Delta E_{bulk,n/bond}^{no}+\Delta E_{bulk,PLCT}^{no}$$

(10) $$\Delta E_{pert,n/bond}^{no}=\sum_{C\in pert}\Delta E^{no}(CL)$$

(11) $$\Delta E_{bulk,n/bond}^{no}=\sum_{A\in bulk}\Delta E^{no}(AL)$$

(12) $$\Delta E_{pert,PLCT}^{no}=\sum_{C\in pert}\Delta E_{eff}^{no}(C)+\Delta E^{no}(L)$$

(13) $$\Delta E_{bulk,PLCT}^{no}=\sum_{A\in bulk}\Delta E_{eff}^{no}(A)$$

For this purpose, the amino acid residues have to be divided between these two regions. The extension of the perturbed region should be appropriately selected to minimize the absolute value of the bulk component ( $\Delta E_{bulk}^{no}$) and of specially its short-range PLCT ( $\Delta E_{bulk,PLCT}^{no}$). Within a hypothetical hybrid (QM/MM) model, it is also essential that the long-range nonbonding bulk energy ( $\Delta E_{bulk,n/bond}^{no}$) can be reproduced by molecular mechanics,

(14) $$\Delta E_{bulk,n/bond}^{no}\approx \sum_{A\in bulk}\sum_{X=N_{A}}^{M_{A}}\sum_{Y=N_{L}}^{M_{L}}\frac{q_{X}q_{Y}}{r_{XY}}+\frac{C_{X,Y}}{r_{XY}^{12}}-\frac{D_{X,Y}}{r_{XY}^{6}}$$

where q_X_ is the charge of atom X, q_Y_ is the charge of atom Y, C_X,Y_ and D_X,Y_ are de van der Waals parameters associated with atoms X and Y, and r_XY_ is the distance between the same atoms. In equation (14), N_A_ and M_A_ are respectively the first and the last atoms of amino acid A. In the same equation, N_L_ and M_L_ have the same meaning for the ligand. In this work, the AMBER99 force field [21] was used to parameterize the bulk terms (14) and the atomic point charges (q_X_ and q_Y_) were calculated using both Mulliken [22] and Merz-Kollman [23] schemes. On the other hand, the effective interaction energy between a residue (C) included in the strongly perturbed region and the ligand has to be calculated at a quantum level.

In this work, the association of CMTI-I and glycerol was studied at an semiempirical level [26], AM1, using the SEEB modified formalism described above. The initial structure of CMTI-I-glycerol complex was obtained from X-ray crystallography with 1.03 Å resolution [24] and can be found in the Protein Data Bank (PDB, 2004) with the reference 1LU0. The geometries of all species (both monomers and complex) were optimized using the MOPAC2002 package [25] in a Pentium 4 computer.

## 3. Discussion

The physically meaningful components of the complex CMTI-I glycerol stabilization energy, obtained using the modified SEEB formalism, are presented in Table 1. The nonbonding term is the dominant component for the stabilization energy. However, the PLCT and the conformational rearrangements components have an important corrective effect.

**Figure 2.**
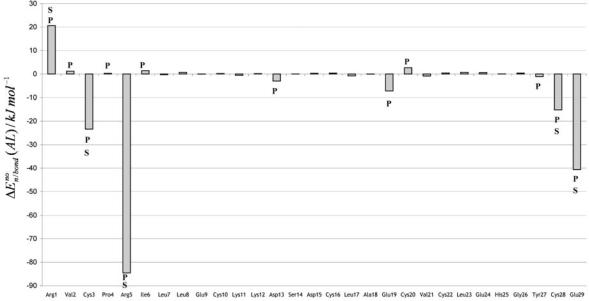
Nonbonding amino acid residue (A)-ligand (L) interaction energies for CMTI-I:glycerol complex. The residues included in the strongly perturbed region are identified by the symbol (P). The residues that strongly interact with the ligand are identified by the symbol (S).

The nonbonding interaction energies between the amino acids and the ligand are presented in Figure 2.

**Figure 3.**
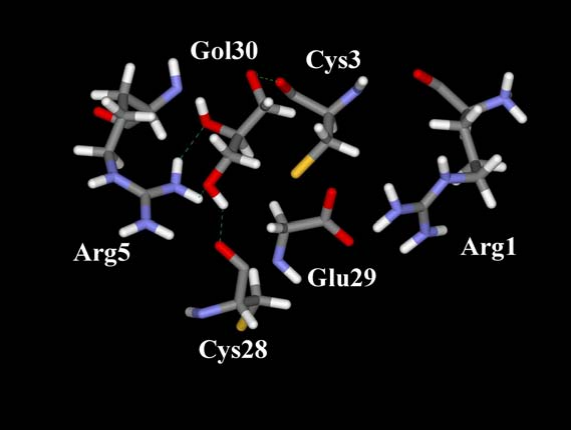
Schematic representation of the of the strongest amino acid residues-ligand interactions (nonbonding interaction energies with absolute values larger than 10 kJ mol^−1^) in the CMTI-I-glycerol complex.

Five residues (Arg1, Cys3, Arg5, Cys28 and Glu29) have the most relevant contributions, which correspond to absolute values larger than 10 kJ mol^−1^. Three residues (Cys3, Arg5 and Cys28) are involved in specific hydrogen bonds with the hydroxyl groups of glycerol (see Figure 3). Arg5 is of particular importance, because this residue establishes two hydrogen bonds of this type and is responsible for approximately 57 % of the nonbonding energy. The two terminal residues (Arg1 and Glu29) are connected by an internal hydrogen bond involving the guanidinium (Arg1) and carboxylate (Glu29) groups.

**Figure 4.**
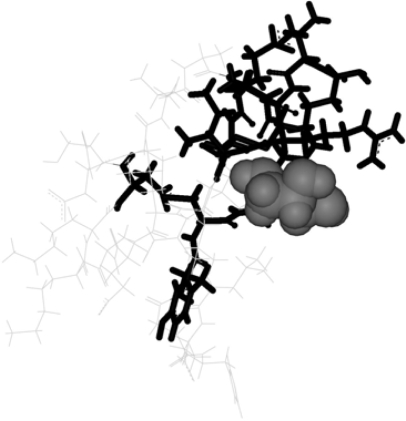
Three dimensional structure of the CMTI-I-glycerol complex. Glycerol is represented in CPK, the strongly perturbed region is represented in black bold and the bulk region is represented in line gray.

**Figure 5.**
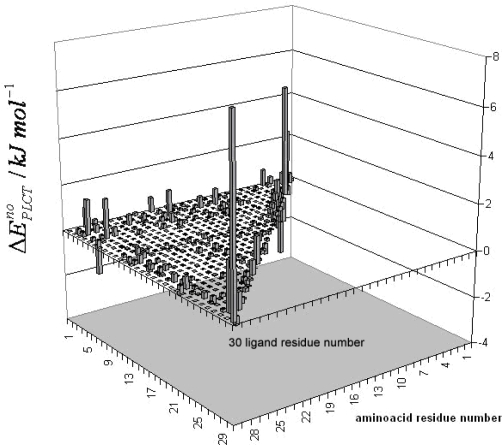
Intra and inter-fragment terms of the polarization plus charge transfer (PLCT) energy. The amino acids are numbered sequentially. The residue 30 corresponds to the glycerol.

In the complex in study, the most electropositive groups of glycerol are oriented in the opposite direction of this guanidinium-carboxylate salt-bridge (see Figures 3 and 4). Therefore, the attractive (Glu29) and repulsive (Arg1) contributions of these residues can be explained by non-specific electrostatic interactions with the ligand. The PLCT energy is represented in Figure 5, as a sum of intra and inter-fragment terms (see equation A9).

**Figure 6.**
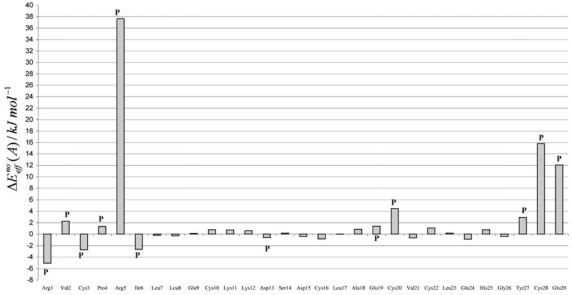
Residue (A) addictive effective PLCT energies. The residues included in the strongly perturbed region are identified by the symbol (P).

Residue addictive effective PLCT terms, defined according to equations (B1) and (B2), are presented in Figure 6. To obtain a bulk region in consistency with the requirements presented in the previous section, all the residues having effective PLCT contributions larger in absolute value than 1.0 kJ mol^−1^ and/or a nonbonding interaction with ligand larger in absolute value larger than 3.0 kJ mol^−1^ were included in the strongly perturbed region. Twelve residues (Arg1, Val2, Cys3, Pro4, Arg5, Ile6, Asp13, Glu19, Cys20, Tyr27, Cys28 and Glu29) satisfied this requirement.

**Figure 7.**
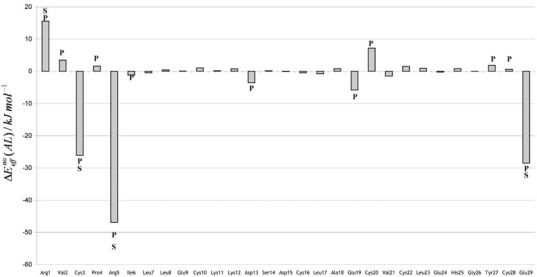
Effective amino acid residue (A)-ligand (L) interaction energies for CMTI-I:glycerol complex. The residues included in the strongly perturbed region are identified by the symbol (P). The residues that strongly interact with the ligand are identified by the symbol (S).

The global contributions of the amino acid residues for the stability of the CMTI-I:glycerol complex was evaluated using the effective pair-wise potential introduced in the previous section. The results obtained are presented in Figure 7. Including these corrective effects, only four residues (Arg1, Cys3, Arg5 and Glu29) were verified to have significant effective contributions (larger than 10 kJ mol^−1^) for the stabilization energy. A fifth residue (Cys28) had been previously identified as relevant, because its nonbonding interaction energy with ligand is markedly negative (–15.2 kJ mol^−1^). However, this residue has an opposite effective PLCT energy of 15.8 kJ mol^−1^ and its overall contribution is near null.

The partition into the strongly perturbed and bulk components is presented in Table 2. These components were evaluated using quantum and classical formalisms. The results obtained enable us to conclude that the interaction energy of bulk region with ligand is well-reproduced by classical potentials, when Merz-Kollman charges are used.

## 4. Conclusions

Using the SEEB formalism, the association of the CMTI-I with glycerol was analysed. The stability of the correspondent complex can be mostly associated with four residues (Arg1, Cys3, Arg5 and Glu29). The residues are also divided between a strongly perturbed and a bulk region. This spatial partition was carried out according to the appropriate requirements previously discussed. In fact, the correspondent short-range bulk component ( $\Delta E_{bulk,PLCT}^{\Gamma }$) is 1.86 kJ mol^−1^ representing only 6.3 % of the total stabilization energy (−29.05 kJ mol^−1^) and the long-range bulk component ( $\Delta E_{bulk,n/bond}^{no}$) is well-reproduced by an AMBER-type potential. In a previous work, it was verified that the Mulliken charges are more appropriate to reproduce such type of potentials in small dimeric species [17]. However, in the globular proteic environment studied in this work, the Merz-Kollman charges seem to be preferable.

The spatial decomposition used in this study constitutes a rational methodology to build QM/MM models. For this purpose, the short-range PLCT term ( $\Delta E_{bulk,PLCT}^{no}$) can be neglected and the long-range nonbonding term ( $\Delta E_{bulk,n/bond}^{no}$) can be calculated at a molecular mechanics level. On the other hand, the strongly perturbed region should be described at an appropriate quantum level.

The modified SEEB method, introduced in this work, enables the description of a protein-ligand association process in terms of a pair-wise interaction potential. This effective potential includes the nonbonding interaction between each pair and the correspondent PLCT correction associated with electronic rearrangement effects. The associated (amino acid-ligand) pair-wise energies can be assumed as physically meaningful components, which can provide an important contribution to better understanding of the protein-ligand association processes.

## Figures and Tables

**Table 1.** Physically meaningful components (kJ mol^−1^) of the stabilization energy for the association between CMTI-I and glycerol.

	$\Delta E_{n/bond}^{no}$	$\Delta E_{PLCT}^{no}$	$\Delta E_{rearr}^{no}$	Δ*E^no^*
**Protein**	-	35.30	33.11	-
**Ligand**	-	33.14	16.89	-
**Total**	−147.50	68.45	50.00	−29.05

**Table 2.** Spatial components (kJ mol^−1^) of the stabilization energy for the association between CMTI-I and glycerol.

$\Delta E_{rearr}^{no}$	$\Delta E_{bulk,PLCT}^{no}$	$\Delta E_{bulk,n/bond}^{no}$			$\Delta E_{bulk}^{no}$	$\Delta E_{pert,PLCT}^{no}$	$\Delta E_{pert,n/bond}^{no}$	$\Delta E_{pert}^{no}$	Δ*E^no^*
		Q	Ml	MK					
50.00	1.86	1.277	0.300	1.347	3.13	66.59	−148.77	-	-

Q: Quantum; Ml-Molecular mechanics with Mulliken charges; Mk: Molecular mechanics with Merz-Kollman charges.

## Appendix A

### Detailed Description of the Physically Meaningful Decomposition

The physically meaningful decomposition, presented in equation (3) to (4) of the second section, is here discussed in detail.

The conformational rearrangement components, $\Delta E_{rearr}^{no}$ (*P*) and $\Delta E_{rearr}^{no}$ (*L*) in equation (3), are defined as,

(A1) $$\Delta E_{rearr}^{no}(P)=E^{\infty }(P_{\left(rear\right)})-E^{\infty }(P)$$

(A2) $$\Delta E_{rearr}^{no}(L)=E^{\infty }(L_{\left(rearr\right)})-E^{\infty }(L)$$

where the superscription (∞) represents a non-binding states where the fragments P and L are at infinite distance. These components are associated with the conformational energy rearrangements occurring in step 1 for the protein (Δ*E^no^* (*P*)) and the ligand (Δ*E^no^* (*L*)) respectively. From a physical point of view, each of these terms represents the energy cost for the correspondent specie (P or L) associated with the transition from the free optimized structure to the free rearranged structure which is the most appropriate for the P:L docking.

(A3) $$P\overset{\Delta E_{rearr}^{no}(P)}{\to }P_{\left(rearr\right)}$$

(A4) $$L\overset{\Delta E_{rearr}^{no}(L)}{\to }L_{\left(rearr\right)}$$

Consequently, the total conformational energy component ( $\Delta E_{n/bond}^{no}$) is calculated as the sum of (A1) and (A2) terms:

(A5) $$\Delta E_{rearr}^{no}=\Delta E_{rearr}^{no}(P)+\Delta E_{rearr}^{no}(L)$$

The PLCT and nonbonding components are associated with step 2, of the hypothetical mechanism presented in Figure 1. In this step, the rearranged fragments are docked, preserving these internal geometries P(rearr) and L (rearr), and originating the final optimized complex (P:L). In this sense, these components have a pure electronic nature, occurring without any modification of the intra-fragment nuclear positions.

The PLCT components are then defined as:

(A6) $$\Delta E_{PLCT}^{no}(P)=E^{no}(P_{\left(rearr\right)})-E^{\infty }(P_{\left(rearr\right)})$$

(A7) $$\Delta E_{PLCT}^{no}(L)=E^{no}(P_{\left(rearr\right)})-E^{\infty }(L_{\left(rearr\right)})$$

Each of these terms represents the electronic energy cost for the correspondent fragment, associated with transition of (P(rearr) or L(rearr)) species from the free rearranged state (∞) to the binding state (no) where the dimeric P:L specie is formed. These terms are originated by polarization plus charge transfer effects, associated with electronic density redistribution within each fragment (P(rear)) or L(rear)) and electronic charge transfer between these species. These components involve energy terms that exist both in free rearranged species (P(rearr)) or L(rearr)) and in the dimer (P:L). However, the associated values are not conserved between these two states due to the mentioned electronic effects. Each of these terms includes the intra-fragment kinetic electronic energy, the attraction energies between the electronic density of the fragment and its nuclei, and the repulsion energy associated with its electronic density.

Considering that the protein (P) is constituted by n amino acids residues, its PLCT component can be partitioned into intra and inter residues terms:

(A8) $$\Delta E_{PLCT}^{no}(P)=\sum_{A=1}^{n}\Delta E^{no}(A)+\sum_{A=1}^{n}\sum_{B=1}^{A-1}\Delta E^{no}(AB)$$

Finally, the total PLCT component can be calculated as:

(A9) $$\Delta E_{PLCT}^{no}=\sum_{A=1}^{n}\Delta E^{no}(A)+\sum_{A=1}^{n}\sum_{B=1}^{A-1}\Delta E^{no}(AB)+\Delta E^{no}(L)=\Delta E_{PLCT}^{no}(P)+\Delta E_{PLCT}^{no}(L)$$

In equation (A9), the terms Δ*E^no^*(*A*) and Δ*E^no^*(*L*) correspond to the energy variations during the mentioned step 2, associated with the amino acid A and the ligand respectively. In the same equation, Δ*E^no^*(*AB*) is associated with the variation in interaction energy between the amino acids A and B during the same step.

The non bonding component $\Delta E_{n/bond}^{no}$ can be calculated as a sum of pair-additive terms,

(A10) $$\Delta E_{n/bond}^{no}=\sum_{A=1}^{n}\Delta E^{no}(AL)$$

where Δ*E^no^* (*AL*)is the interaction energy between the amino acids A and the ligand (L) that only exists in the dimer. This term includes the attraction energies between the electronic density of A and the nuclei of L, the attraction energies between the electronic density of L and the nuclei of A, the repulsion energy between the electronic densities of A and L, and the repulsion energies between the nuclei of the two species.

## Appendix B

### Detailed description and justification of the pair-wise decomposition

The pair-wise decomposition, presented in equation (5) of the second section, is here discussed and justified in detail. Analyzing the equation (A9) in Appendix A, it is obvious that the PLCT component is not that residue-additive due the occurrence of the inter-residue terms (Δ*E^no^*(*AB*)) . However, effective additive terms can be obtained dividing equally these interaction energies by the two partner residues (A and B) involved:

(B1) $$\Delta E_{PLCT}^{no}=\sum_{A=1}^{n}\Delta E_{eff}^{no}(A)+\Delta E^{no}(L)$$

With

(B2) $$\Delta E_{eff}^{no}(A)=\Delta E^{no}(A)+\sum_{B\neq A=1}^{n}\Delta E^{no}(AB)/2$$

For the non-covalent interactions, the simplification adopted in equations (B1) and (B2) is a natural criterion to divide the inter-residue interactions energies. For covalent interactions, this corresponds to a Mulliken-like approach that involves some degree of arbitrariness. However, in general, the methodology proposed here seems to be a reasonable approach to obtain effective PLCT terms.

An effective interaction potential between the ligand L and an amino acid (A) ( $\Delta E_{eff}^{no}$(*AL*)), combining non-bonding and PLCT contributions, can now be obtained from equations (A10), (B1) and (B2). This potential evaluates the nonbonding interaction between A and L, corrected by the PLCT reorganization associated with both species.

(B3) $$\Delta E_{eff}^{no}(AL)=\Delta E^{no}(AL)+\Delta E_{eff}^{no}(A)+\alpha_{AL}\times \Delta E^{no}(L)$$

In equation (B3), the coefficient (*α_AL_* ) is defined as,

(B4) $$\alpha_{AL}=\Delta E^{no}(AL)/\Delta E^{no}$$

The mutual PLCT energy cost of two residues (A and L) is clearly correlated with the magnitude of the correspondent interaction. In fact, if the A-L interaction is extremely weak their mutual polarization effects can be neglected. On the other hand, if the amino acid A strongly interacts with L these polarization effects should be very important. Almost all PLCT effect induced on amino acid A is originated, directly or indirectly, by the interaction with the ligand. On the other hand, the PLCT effects in the ligand are induced by its interactions with the amino acids of the protein (P). In this context, the coefficient (*α_AL_* ) can be considered as a good weighting factor to evaluate the degree of contribution of the A-L interaction to the PLCT ligand energy cost.
